# Reconsidering the usefulness of long-term high-dose buprenorphine

**DOI:** 10.3389/fpsyt.2024.1401676

**Published:** 2024-07-23

**Authors:** Lakshit Jain, Thomas W. Meeks, Christopher K. Blazes

**Affiliations:** ^1^ Department of Psychiatry, University of Connecticut, Farmington, CT, United States; ^2^ Department of Psychiatry, Oregon Health & Science University, Portland, OR, United States

**Keywords:** nitazenes, safety, opioid use disorder (OUD), fentanyl, buprenorphine (BN), high dose, high potency synthetic opioids, prescription opiates

## Abstract

Buprenorphine has been successfully used for decades in the treatment of opioid use disorder, yet there are complexities to its use that warrant attention to maximize its utility. While the package insert of the combination product buprenorphine\naloxone continues to recommend a maximum dose of 16 mg daily for maintenance, the emergence of fentanyl and synthetic analogs in the current drug supply may be limiting the effectiveness of this standard dose. Many practitioners have embraced and appropriately implemented novel practices to mitigate the sequelae of our current crisis. It has become common clinical practice to stabilize patients with 24 - 32 mg of buprenorphine daily at treatment initiation. Many of these patients, however, are maintained on these high doses (>16 mg/d) indefinitely, even after prolonged stability. Although this may be a necessary strategy in the short term, there is little evidence to support its safety and efficacy, and these high doses may be exposing patients to more complications and side effects than standard doses. Commonly known side effects of buprenorphine that are likely dose-related include hyperhidrosis, sedation, decreased libido, constipation, and hypogonadism. There are also complications related to the active metabolite of buprenorphine (norbuprenorphine) which is a full agonist at the mu opioid receptor and does not have a ceiling on respiratory suppression. Such side effects can lead to medical morbidity as well as decreased medication adherence, and we, therefore, recommend that after a period of stabilization, practitioners consider a trial of decreasing the dose of buprenorphine toward standard dose recommendations. Some patients’ path of recovery may never reach this stabilization phase (i.e., several months of adherence to medications, opioid abstinence, and other clinical indicators of stability). Side effects of buprenorphine may not have much salience when patients are struggling for survival and safety, but for those who are fortunate enough to advance in their recovery, the side effects become more problematic and can limit quality of life and adherence.

## 1 Introduction

With fentanyl, nitazenes, and other High Potency Synthetic Opioids (HPSOs) worsening the opioid overdose death crisis (1), clinicians in the field have needed to rapidly adapt their clinical practices and utilize strategies prior to supportive evidence of their safety and efficacy. “Classic” buprenorphine initiations with buprenorphine target doses of 16 mg/day are often ineffective in stabilizing patients with HPSO use, who seem to have exaggerated tolerance compared to those using heroin and prescription opioids. Buprenorphine doses as high as 64 mg/day have been reported to manage these emerging clinical challenges. For the purpose of this discussion, we include 16 mg/day as standard dosing and not “high dose,” although some publications define “higher dose” as ≥16 mg/day.

There are no formal guidelines to support the use of high-dose buprenorphine (HDB, hereafter defined as >16 mg/day), and there is limited support for this practice in the literature. The 2020 focused update of the American Society of Addiction Medicine (ASAM) national practice guidelines mentions that higher doses of buprenorphine (≥16 mg/day) can be more effective than lower doses (2). They also point out that there is limited evidence of efficacy of doses >24 mg/day, and that higher doses may increase the risk of diversion (3). A meta-analysis by Fareed et al. (3) identified that 16-32 mg/day buprenorphine predicts increased adherence to treatment, which was further supported by a Cochrane review in 2014 (4). Furthermore, Grande et al. argued that the FDA erred by tightening the maximum dosing guidance in the buprenorphine/naloxone package insert in 2010 by stating that “doses higher than 24 mg have not been demonstrated to have a clinical advantage in the treatment” of OUD; and that FDA perpetuates this error by keeping this language in the 2022 package insert despite the significant increase in HPSO related mortality. To support their argument, they (5) discuss how Substance Abuse and Mental Health Services Administration (SAMHSA) 2004 recommendations endorsed buprenorphine dosing up to 32mg/day based on available evidence. The authors highlighted the lifesaving nature of high-dose buprenorphine by citing a 2022 study that found that 5ng/mL steady state plasma concentration (corresponding to 32mg/day sublingual dose) was able to protect against the respiratory depression caused by fentanyl in opioid tolerant participants. They also mention that a 2022 study on buprenorphine extended-release injections found that a 300 mg monthly dose (yielding average plasma concentrations higher than 32mg/day sublingually) increased the likelihood of abstaining from opioids compared to 100 mg/month (5). Additionally, they discussed how buprenorphine diversion induced harm is rare, that buprenorphine is often diverted to self-treat when legal access is unavailable, and that experienced clinicians see diversion as a cost to be weighed against known benefits. The manuscript concluded by asking FDA to correct the label language to promote individualization of buprenorphine up to and > 32mg/day if needed and highlighted that strict dose limits (<32mg/day) harm patients in the context of widespread HPSO use (5).

In 2023, Weimer et al. compiled an ASAM clinical considerations document, specifically addressing treatment for individuals using HPSO (6). While the article does not propose clinical practice guidelines, the article was written by an expert group who performed a narrative review, and a separate group of experts served as a peer review panel. The authors mentioned that buprenorphine should be initiated at a dose of ≥8 mg in patients who may face delays accessing buprenorphine, or if there is a concern for Opioid Withdrawal Syndrome (OWS), likely due to chronic use of HPSO or lack of response to standard buprenorphine initiation (6). They asked clinicians to consider >24 mg/day buprenorphine for mild to moderate OWS during buprenorphine initiation in patients with high opioid tolerance. They also mentioned that some patients with high opioid tolerance may require >24 mg buprenorphine dose during treatment stabilization, but that these higher doses should be reassessed as the patient enters long-term treatment (6).

Despite organizations either declining to recommend (FDA) or showing measured consideration of supporting (ASAM, SAMHSA) HDB, there is growing advocacy by front-line clinicians and subject matter experts to use HDB in persons using HPSOs in the face of increased opioid overdose deaths in this population.

### 1.1 Potential concerns of high-dose buprenorphine

While it is important to take bold steps to save lives immediately, clinicians must also contemplate the long-term impact of their decisions. HDB may be useful in the short term, but this does not appear to be consistently reflected in clinical outcomes. While some RCTs support the utility of HDB (7, 8), a systematic review (of studies that did not involve Fentanyl/HPSO use) did not find evidence that receiving higher than 16 mg daily SL buprenorphine confers added opioid receptor blockade benefits (9). Additionally, available research has not found fentanyl use (as opposed to less potent opioid use) to be clearly related to treatment retention, suggesting that other factors that tend to cluster with fentanyl use, such as psychosocial instability and stimulant use, may explain some of the treatment engagement difficulties often attributed to fentanyl potency (10–13). Thus, further research is needed to verify whether maintaining patients on HDB has clinical utility.

While many patients with OUD may benefit from the acute use of HDB, continuing these doses during the maintenance phase may introduce some deleterious consequences. Although in most circumstances buprenorphine is a remarkably safe medication, there are potential side effects and complications that may be dose dependent. These include constipation, nausea, dental erosion, and, infrequently, adrenal insufficiency (4). One of the authors of this commentary (CB) identified in their clinical practice that HDB specifically causes hyperhidrosis, decreased libido and fatigue that diminish as the dose is decreased. To our knowledge, there is a paucity of examination of dose-related side effects in the literature, and further research is needed. Additionally, HDB can make the management of severe acute pain or emergency surgery more problematic by creating a need for high doses of full agonist opioids for analgesia or sedation (14)

Although buprenorphine is a partial agonist, it can in some circumstances have reinforcing effects at initiation and during dose increases. Jasinski et al. in their 1978 study (15) observed that both acute and chronic administration of buprenorphine led to a “morphine like” euphoria which can last up to 3 days. Subjects of this study were young, healthy male prisoner volunteers, with “histories of recurrent addiction to narcotics,” but at the time of the study, none were physically dependent on opioids (15). In our clinical practice, we (CB) commonly observe patients on buprenorphine experiencing a brief (up to 5 day long) period of euphoria following an increase in their dose. Many patients and/or clinicians can misinterpret these feelings as symptom relief or reduction in cravings, and this scenario can lead to progressive increases in doses over time in patients trying to re-experience euphoric reinforcement.

### 1.2 Pharmacological challenges with using high dose buprenorphine

HDB may be associated with underappreciated pharmacological issues, such as adverse effects, impacts of active metabolites, and interactions with other medications. While buprenorphine’s safety profile for respiratory depression relative to full agonists is established, several cases of deaths secondary to asphyxia have been reported from intravenous misuse of buprenorphine (16, 17). Although buprenorphine-related overdose deaths are often attributed to concomitant use of benzodiazepines and other sedatives, Seldén et al, reported that 10% of buprenorphine-related deaths did not involve other intoxicants, suggesting buprenorphine alone can still lead to fatal respiratory depression (17). A recent review by White et al. observed that buprenorphine used in management of acute pain in opioid nontolerant patients exhibited significantly greater respiratory depression than morphine in 4 out of 19 studies They reported that buprenorphine did not alter tidal volume but slowed respiratory rate and concluded that buprenorphine exhibited a level of respiratory depression similar to morphine, albeit in a sample than did not use HPSOs (18). Some patients with OUD have inconsistent access to opioids and can lose their tolerance. For these individuals, even buprenorphine could in certain instances cause overdose. This is concerning, as buprenorphine has a high affinity for the mu opioid receptor that is difficult to reverse with naloxone.

Another concern with using HDB is that the primary metabolite of buprenorphine, norbuprenorphine, is a full agonist at the mu and a partial agonist at the kappa opioid receptor (19). Norbuprenorphine and buprenorphine in animals had similarly potent binding affinities to mu and kappa receptors, suggesting norbuprenorphine could compete with buprenorphine in binding to these receptors (19). Norbuprenorphine does not have a ‘ceiling effect’ on respiratory depression and appears to have a respiratory depressant effect 10 times that of buprenorphine in rat models. However, norbuprenorphine has significant affinity for P-glycoprotein (an efflux transporter that moves harmful compounds from the central nervous system back into the bloodstream) and hence cannot penetrate the blood brain barrier effectively. The metabolite thus does not pose a risk to patients on standard doses of buprenorphine (20). However, a case report observed that norbuprenorphine was present in the brain after a large buprenorphine overdose, highlighting that the P-glycoprotein system can be overwhelmed and that chronic HDB might thus increase overdose risk (7).

Norbuprenorphine is also thought to cause opioid induced constipation via mu-receptors in the gastrointestinal (GI) system. This was supported by a 2016 study examining why sublingual buprenorphine–naloxone was associated with higher rates of constipation than buccal formulations (21). They used a new buccal formulation with a backing film that forced buprenorphine absorption only through oral mucosa and prevented the GI absorption/first-pass metabolism, that is somewhat unavoidable with sublingual formulations. Using the backing film, projected steady state plasma norbuprenorphine concentrations were nearly 30% lower for buccal compared to sublingual administration. After sublingual administration, norbuprenorphine was about 30% higher than steady-state buprenorphine concentrations (21). Hence, HDB may expose GI receptors to more norbuprenorphine and increase rates of constipation. Norbuprenorphine may also play a role in HDB-associated nausea.

Both buprenorphine and norbuprenorphine are also converted into the active metabolite’s buprenorphine-3-glucuronide and norbuprenorphine-3-glucuronide, respectively. Initially considered to be inactive (7), both glucuronide metabolites were later demonstrated instead to be active. Buprenorphine-3-glucuronide exhibits affinity towards mu, delta and nociceptin receptors, and norbuprenorphine-3-glucuronide exhibits affinity towards kappa and nociceptin receptors (22). The intrinsic activity of the glucuronide metabolites at these receptors is not yet known (20). While buprenorphine-3-glucuronide acts similarly to buprenorphine and thus poses less risk for respiratory depression or sedation, norbuprenorphine-3-glucuronide can cross the blood-brain barrier and cause sedation and reduction in tidal volume (20). Tidal volume reduction in combination with norbuprenorphine’s reduction of respiratory rate may be especially concerning (22). Another potential adverse effect of HDB-associated metabolite elevations is that both norbuprenorphine and norbuprenorphine-3-glucuronide exert a partial kappa agonist effect (19, 22). Buprenorphine is proposed to have potential antidepressant and anxiolytic properties due to its kappa antagonism (23), so opposing metabolite actions could in contrast increase anxiety and depression and contribute to non-adherence and drug use (23).

Another consideration when using HDB is drug-drug interactions. For example, concomitant use of prescribed or non-prescribed benzodiazepines and alcohol increases the likelihood of a fatal overdose (17, 24). Mégarbane et al. postulated that both pharmacodynamic and pharmacokinetic interactions between buprenorphine and benzodiazepines may play a role (16). Gabapentinoids in combination with buprenorphine also increase the risk of respiratory depression and overdose (25). With high psychiatric comorbidity in persons with OUD, other medications such as antipsychotics, hypnotics, and sedating antidepressants are not uncommon and may add to the overdose risk burden (26). Polysubstance use is also common in persons using HPSO, and other emerging novel psychoactive substances such as nitazenes, “designer” benzodiazepines and the high-potency alpha-2 adrenergic receptor agonist xylazine may also increase overdose risk in combination with buprenorphine (1) (27).

### 1.3 Hormonal effects of using high dose buprenorphine

While buprenorphine has less impact on sexual desire and serum testosterone levels than methadone (28), case reports have linked sexual dysfunction, hypogonadism and decline in bone density levels to buprenorphine use (29). Insufficient research has been conducted to identify potential long-term impacts of buprenorphine, including any dose correlations, on hypothalamic-pituitary functions including cortisol and sex hormone regulation (29). Two studies found no correlation between serum testosterone levels and the dose or duration of buprenorphine. Nonetheless, elevated rates of sexual dysfunction have been reported with buprenorphine and might involve mechanisms other than sex hormone suppression (30) Although testosterone supplementation is possible for patients experiencing hypogonadism in order to continue buprenorphine treatment, testosterone supplementation can lead to increased behavioral and sexual impulsivity, aggression and dysphoric mood (31–33). Exogenous testosterone can also suppress endogenous testosterone production and lead to testicular atrophy, infertility, and a permanent reduction in testosterone production.

### 1.4 Increased risk of diversion

Although diversion is a minor concern when taking into account the lifesaving effects of buprenorphine, it is still worth attention. Buprenorphine has monetary value in the drug market and at times is diverted (34, 35). HDB could increase the risk of diversion, as such patients may have successfully experimented with self-decreasing their dose, creating opportunity to divert the remaining supply. One study identified that patients divert buprenorphine both altruistically to help friends (58.9%), and to sell it for monetary gain (57%). Moreover, 27.1% of patients shared their dose as they “didn’t need the whole dose.”

## 2 Discussion

In summary, although still off-label and not tested thoroughly in the literature, buprenorphine given at a dose of 16 mg/day or more is likely indicated in the initial induction and stabilization phase for patients who have been using high potency opioids such as fentanyl. After the stabilization phase (which may never happen for some), the best balance of safety and efficacy may be a dose decrease to the package insert maximum recommended daily dose of 16 mg/day rather than maintaining HDB therapy. This should be conducted in a patient-centered manner that may require ongoing collaboration, gradual dose reduction, and/or resumption of a higher dose if there is clinical destabilization during a dose reduction. A dose reduction may protect patients from experiencing short- and long-term side effects and result in increased medication adherence, both by reducing self-discontinuation due to side effects and by decreasing temptation to divert the clinically superfluous portions of HDB. In some circumstances we have found some patients do even better when maintained at lower doses (8-12 mg/day). Our clinical experience has been that many patients tolerate dose tapers from 24 mg to 16 mg/day after a stabilization period without problematic withdrawal symptoms or worsening cravings.

Despite buprenorphine’s deserved reputation as a relatively low-risk medication due to its mu receptor partial agonism, this reputation is in large part predicated on data and experience treating pre-fentanyl era OUD with more modest doses. HDB may have risks that haven’t been adequately studied. Buprenorphine pharmacokinetics may change in important ways at higher doses, including elevated blood and/or CNS levels of active and more toxic metabolites. Several medication interactions may become more relevant with these higher levels of buprenorphine and its metabolites. Commonly used prescription medications and non-medical substances with respiratory depressant effects may have enhanced synergistic toxicity with such metabolites. Perioperative management and emergency pain treatment could become further complicated. Additional research on the pharmacokinetics and pharmacodynamics of these active metabolites is needed, including more human and/or *in vivo* data involving patients on HDB, to better inform risk-benefit decisions in the long-term treatment of OUD.

Buprenorphine is an effective tool in the treatment of OUD (36), and recently the federal government reinforced its utility by easing restrictions on prescribing by dropping the X-waiver requirement. Clinicians with a standard DEA registration can now prescribe buprenorphine for opioid use disorder. Broadening access to treatment for OUD is an important public good, and provider anxiety about its use can be exaggerated and a barrier to treatment. At the same time, with many new clinicians gaining access to prescribe buprenorphine, better guidelines are needed to help less experienced clinicians navigate some of the above-described nuances and know when to refer to an addiction specialist. While it is important that buprenorphine be available for patients with opioid use disorders, clinicians should still be cognizant of the potential side effects, complications, and diversion of buprenorphine.

## 3 Conclusion

Buprenorphine is a safe medication and has minimal side effects when taken at approved doses. The addiction field should continue to strive for high-quality data that clarifies the relative risk-benefit profile of the long-term use of high dose buprenorphine for OUD associated with high potency synthetic opioids, which is becoming increasingly practiced as the standard of care for such patients. High-dose, long-term off-label use of buprenorphine has the potential to lead to higher side effect burden, safety concerns, and diversion. HDB can be an effective tool for the induction and stabilization phases of treatment for patients with OUD using HPSOs. Clinicians should, however, periodically review the utility and necessity of maintaining high-dose buprenorphine for patients who have demonstrated stability in their recovery. The burden of buprenorphine side effects may carry greater weight for patients in advanced stages of recovery.

## Data availability statement

The original contributions presented in the study are included in the article/supplementary material, further inquiries can be directed to the corresponding author/s.

## Author contributions

LJ: Writing – original draft, Writing – review & editing. TM: Writing – original draft, Writing – review & editing. CB: Writing – original draft, Writing – review & editing.
